# Role of Uremic Toxins, Oxidative Stress, and Renal Fibrosis in Chronic Kidney Disease

**DOI:** 10.3390/antiox13060687

**Published:** 2024-06-03

**Authors:** Weronika Frąk, Bartłomiej Dąbek, Marta Balcerczyk-Lis, Jakub Motor, Ewa Radzioch, Ewelina Młynarska, Jacek Rysz, Beata Franczyk

**Affiliations:** 1 Department of Nephrocardiology, Medical Univeristy of Lodz, ul. Zeromskiego 113, 90-549 Lodz, Poland; 2 Department of Nephrology, Hypertension and Family Medicine, Medical University of Lodz, ul. Zeromskiego 113, 90-549 Lodz, Poland

**Keywords:** chronic kidney disease, uremic toxins, oxidative stress, renal fibrosis, treatment

## Abstract

Affecting millions of people worldwide, chronic kidney disease is a serious medical problem. It results in a decrease in glomerular filtration rate below 60 mL/min/1.73 m, albuminuria, abnormalities in urine sediment and pathologies detected by imaging studies lasting a minimum of 3 months. Patients with CKD develop uremia, and as a result of the accumulation of uremic toxins in the body, patients can be expected to suffer from a number of medical consequences such as progression of CKD with renal fibrosis, development of atherosclerosis or increased incidence of cardiovascular events. Another key element in the pathogenesis of CKD is oxidative stress, resulting from an imbalance between the production of antioxidants and the production of reactive oxygen species. Oxidative stress contributes to damage to cellular proteins, lipids and DNA and increases inflammation, perpetuating kidney dysfunction. Additionally, renal fibrogenesis involving the accumulation of fibrous tissue in the kidneys occurs. In our review, we also included examples of forms of therapy for CKD. To improve the condition of CKD patients, pharmacotherapy can be used, as described in our review. Among the drugs that improve the prognosis of patients with CKD, we can include: GLP-1 analogues, SGLT2 inhibitors, Finerenone monoclonal antibody—Canakinumab and Sacubitril/Valsartan.

## 1. Introduction

Chronic kidney disease (CKD) is a progressive condition characterized by the gradual deterioration of kidney function over an extended period. It creates a significant public health challenge worldwide, affecting millions of individuals and often leading to serious complications if left untreated [1,2]. CKD is a condition characterized by a dysfunction of the kidneys or structural abnormalities thereof. It is defined as a glomerular filtration rate (GFR) of less than 60 mL/min/1.73 m, or by the presence of renal marker damage, including albuminuria, urine sediment abnormalities, electrolyte, and other abnormalities due to tubular disorders, structural abnormalities discovered by histology, or imaging and history of kidney transplantation, or both, of at least 3 months’ duration, according to the KDIGO guidelines. CKD can be recognized without acknowledgment of its cause [3].

Several factors contribute to the development and progression of CKD. Hypertension, diabetes mellitus, cardiovascular diseases (CVD), obstructive sleep apnea, or episodes of acute kidney injury have been linked to the development of CKD. Other risk factors include smoking, excessive alcohol consumption, obesity, genetics, age, certain medications (such as NSAIDs and antibiotics), as well as recurrent kidney stones or urinary tract obstructions. It is noteworthy that some of these risk factors are modifiable, offering opportunities to delay or prevent the advancement of kidney failure [3,4,5,6].

CKD is classified into stages based on the severity of kidney damage and the level of kidney function, ranging from stage 1 (mildest) to stage 5 (most severe), based on eGFR level [3].

CKD involves complex molecular mechanisms, including oxidative stress, fibrosis, and dysregulation of various signaling pathways. These processes contribute to the progressive decline in kidney function, eventually leading to tissue damage and impaired renal function. This review aims to describe new insights into the molecular mechanism of CKD. We focused on uremic toxins, oxidative stress, renal fibrosis, and new treatment targets that have emerged as knowledge about these mechanisms has developed. The combined findings from both basic scientific research and clinical studies offer valuable insights into the precise mechanisms underlying CKD. Further investigation is warranted to advance our knowledge of the pathogenesis of CKD and establish novel treatments and preventive strategies.

## 2. Uremic Toxins

Uremic toxins (UTs) are harmful substances that are removed from the body by the kidneys under physiological conditions [7]. In patients with CKD, the removal of UTs is limited, leading to uremia, which is a life-threatening condition [7,8]. Even in patients receiving dialysis therapy, it is not possible to remove all UTs that accumulate in the body, contributing to the progression of CKD and increasing the risk of cardiovascular events [8]. Accumulation of toxins in patients suffering from renal failure is not only due to their reduced excretion due to reduced eGFR. Also contributing to the accumulation of toxins in the body are intestinal microbial imbalances, which correspond with the widening of the spectrum of UTs. In addition, renal failure is accompanied by local inflammation, which promotes increased production of cytokines and receptors being a factor in the accumulation of medium molecules [9].

**Figure 1:**
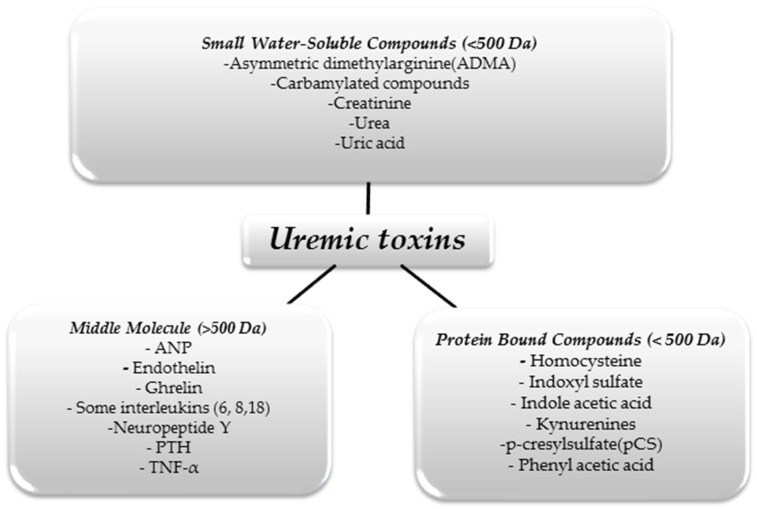
Classification of uremic toxins [10].

We can divide UTs in terms of their size and physicochemical properties. This division is shown in Figure 1 [10].

The substances shown in the figure above are just some examples of UTs, as it is assumed that there are about 150 different molecules that constitute this group [11]. As we mentioned above, only some of these molecules can be effectively removed using renal replacement therapies. With hemodialysis, small water-soluble molecules can be eliminated from the body. However, despite continuous improvements in extracorporeal blood purification techniques, large protein-linked molecules that often have toxic effects on the body remain a problem. For example, in uremia, there is an increase in pro-inflammatory interleukins affecting, among other things, the development of atherosclerotic lesions, thus increasing mortality from cardiovascular incidents in people with CKD [12].

Other molecules that are problematic to eliminate from the body during dialysis are protein-linked: indoxyl sulfate (IS) and indole-3-acetic acid (IAA). They belong to the group of indole UTs formed from the metabolic transformation of dietary tryptophan. In the indole pathway, thanks to the intestinal microbiota, tryptophan is converted into indole, which is absorbed into the bloodstream. In the next step, it is metabolized in the liver, where it is oxidized and sulfated by the enzyme CYP2E1, cytochrome p450, and sulfatase ultimately forming IS. In contrast, IAA is metabolized from tryptophan directly in the intestines before tryptamine [13]. Physiologically, IS is absorbed in the proximal tubules and then excreted from the body with urine. As renal function in the body weakens, IS levels increase, which is a factor in the progression of CKD [8]. It has also been observed that patients with CKD develop endothelial dysfunction, which is associated with nitric oxide (NO) deficiency. Tumur et al. showed in their study that IS inhibits NO production, which led to the conclusion that increased levels of IS indirectly contribute to endothelial dysfunction and consequently to the development of atherosclerosis in CKD patients [14]. Studies in rats have shown the effect of IS not only on vascular lesions, but have also shown that it contributes to tubulointerstitial damage and consequently to fibrosis of fragments or the entire kidney increasing uremia-induced mortality [15]. As early as in the 1960s, observing the negative effects of IS on the body, studies began to investigate whether the concentration of this substance increases with an increase in dietary tryptophan. These studies confirmed the effect of a diet rich in tryptophan (contained mainly in high-protein foods such as red meat, poultry, seeds and nuts, eggs, and fish) on increased IS production [16].

Another molecule derived from the breakdown of tryptophan, in whose metabolism the gut microbiota plays an important role, is IAA. IAA has been shown to have pro-inflammatory and pro-apoptotic effects, additionally increasing the risk of thrombotic events [17]. In addition, it has been found that high levels of IAA found in CKD patients correlate with an increased rate of major cardiovascular events and increased mortality in this group of uremic patients [18].

Homocysteine (Hcy), which is an amino acid containing a thiol group derived from the breakdown of methionine, also belongs to the group of UTs linked to proteins. Supplied by the diet or derived from the breakdown of endogenous proteins, methionine is converted to S-adenosylmethionine (SAM) thanks to enzymatic reactions, and it is converted to S-adenosylhomocysteine (SAH), from which adenosine and Hcy are formed with the help of SAH hydrolase. The metabolism of Hcy occurs in the trans-sulfuration and remethylation pathways (the cofactors vit. B12 and folic acid are required in this pathway). The trans-sulfuration pathway has been shown to occur mainly in the kidneys, and its defect in sychronic renal failure (ESRD) contributes to increased plasma Hcy concentrations [19]. A study by Cohen et al. showed a relationship between Hcy levels and the severity of CKD. They noted that plasma Hcy levels increased as patients’ GFR decreased [20]. On the other hand, the China Stroke Prevention Primary Prevention Trial (CSPPT) noted that low-dose folic acid (0.8 mg/day), as a cofactor required for homocysteine in the remethylation pathway, reduced plasma HCy levels. Further studies should be considered to see if low doses of folic acid can limit the progression of CDK development [21]. When considering how high levels of Hcy in CKD patients affect the body, it was noted that it disrupts cellular homeostasis and negatively affects redox reactions, thereby increasing oxidative stress [22]. In addition, a study conducted on rodents also showed that Hcy increases oxidative stress and furthermore stimulates the expression of pro-inflammatory cytokines, such as tumor necrosis factor α (TNFα) and interleukin 6 (IL-6) [23].

In conclusion, renal failure is one of the main causes of UTs accumulation and the development of uremia in CKD patients [7]. Some studies have shown that the kidneys are not the only organ regulating the level of UTs in the body, and the gut and intestinal microflora also play an important role [24]. In addition to uremic symptoms, as a result of increased oxidative stress, UTs contribute to the progression of CKD through renal hypoxia resulting from increased oxygen consumption [25]. In addition, increased oxidative stress in patients with CKD disrupts the balance between bone formation and bone resorption, thus predisposing patients to more frequent fractures [26]. Some data have shown that a low-protein diet and probiotics or prebiotics by affecting the intestinal microflora can reduce UTs accumulation even in CKD patients. However, more studies are needed to confirm this thesis [27].

## 3. Oxidative Stress

Oxidative stress, caused by an imbalance between the generation of ROS and the production of antioxidants, plays a pivotal role in the pathogenesis and progression of CKD [28]. ROS are highly reactive molecules that include free radicals [29]. In CKD, the imbalance between ROS production and antioxidant defense mechanisms leads to oxidative stress, causing damage to cellular components including lipids, proteins, and DNA [30]. This oxidative damage further exacerbates inflammation, creating a cycle of oxidative stress and inflammation that perpetuates renal injury and dysfunction [31].

Chronic inflammation is one of the main indicators of CKD and a significant cause of oxidative stress. In response to injury, immune cells release pro-inflammatory cytokines and chemokines, such as TNFα, Il-6, and Il-1 beta. These inflammatory mediators activate ROS-producing enzymes, including nicotinamide adenine dinucleotide phosphate (NADPH) oxidase, xanthine oxidase, and inducible NO synthase, leading to increased ROS generation within renal cells [32,33]. Moreover, inflammatory cells, such as macrophages and neutrophils, produce ROS as part of their antimicrobial defense mechanisms. However, a sustained inflammatory state contributes to the excessive generation of ROS; thus, it can damage surrounding tissues, exacerbating the renal injury, inflammation, and disease progression [32,34]. Moreover, oxidative stress promotes the activation of nuclear factor-kappa B (NF-κB) and other transcription factors involved in inflammatory gene expression. NF-κB is a key regulator of the inflammatory response and is activated in response to ROS-induced cellular damage. The activation of NF-κB leads to the transcription of pro-inflammatory genes, perpetuating the inflammatory cascade and amplifying renal injury in CKD [35,36,37].

Ischemia-reperfusion injury (IRI) occurs when blood flow to the kidneys is temporarily interrupted, followed by the restoration of blood flow [38]. IRI is a common feature of acute kidney injury (AKI), a major risk factor for the development and progression of CKD [39]. Further, IRI generates ROS through various mechanisms, including the activation of xanthine oxidase, mitochondrial dysfunction, and the release of inflammatory mediators such as cytokines and chemokines [40]. During ischemia, the lack of oxygen and nutrients leads to impaired mitochondrial function and adenosine triphosphate (ATP) depletion, resulting in ROS accumulation. Subsequent reperfusion exacerbates oxidative stress by promoting the production of additional ROS, particularly through the activation of xanthine oxidase and the electron transport chain in mitochondria. The resultant oxidative stress damages renal cells, including tubular epithelial cells, endothelial cells, and interstitial cells, leading to tissue injury and dysfunction [41,42].

Mitochondria are central organelles involved in energy production, ROS generation, and apoptosis [43]. Mitochondrial dysfunction has been recognized as a contributor to many diseases. Ho et al. indicated that oxidative stress injury can be caused by impaired mitochondria with excessive levels of ROS in CKD [44]. Several factors, including uremic toxins, metabolic dysregulation, and impaired mitochondrial biogenesis, can disrupt mitochondrial function and exacerbate ROS production. It showed that oxidative stress and renal failure triggered by these risk factors damaged worsened oxidative injury because of an imbalance in mitochondrial antioxidants and the ensuing enlargement of the mitochondria [44,45]. Impaired oxidative phosphorylation and electron transport chain dysfunction lead to the leakage of electrons and the generation of superoxide radicals [46]. Furthermore, mitochondrial DNA damage and mutations can impair mitochondrial function and exacerbate oxidative stress. The resultant ROS production contributes to cellular damage and apoptosis, further impairing renal function and exacerbating CKD progression [47].

**Figure 2:**
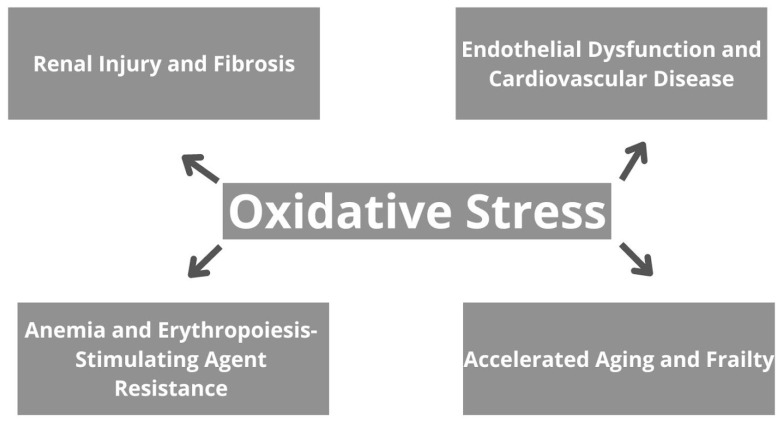
The effects of oxidative stress in chronic kidney disease.

The consequences of oxidative stress in CKD are broad and encompass various aspects of renal pathology, as well as systemic complications [48]. To begin with, oxidative stress contributes to renal injury and fibrosis by inducing apoptosis, necrosis, and inflammation in renal cells, therefore leading to tissue damage and dysfunction, and further progressive decline in kidney function [48,49]. Moreover, oxidative stress promotes endothelial dysfunction, atherosclerosis, and CVD in CKD patients. It impairs NO bioavailability, promotes vasoconstriction, and increases vascular permeability, contributing to hypertension and cardiovascular complications [50]. Oxidative stress also plays a role in the pathogenesis of anemia in CKD by suppressing erythropoietin production and impairing iron utilization in erythropoiesis. Furthermore, it contributes to erythropoiesis-stimulating agent resistance, limiting the efficacy of anemia management strategies [51,52]. Additionally, oxidative stress-induced cellular senescence, DNA damage, and mitochondrial dysfunction accelerate aging and frailty in CKD patients. Skeletal muscle wasting, fatigue, and decreased functional capacity are common manifestations of frailty, further compromising quality of life and increasing morbidity and mortality [53]. Generally, oxidative stress in CKD contributes to renal and systemic complications, highlighting the importance of targeted therapeutic interventions to mitigate its impact and improve patient outcomes. Implications of oxidative stress on CKD are summarized in Figure 2.

Understanding the mechanisms underlying oxidative stress in CKD is crucial for developing targeted therapeutic interventions to lessen renal injury and improve patient outcomes. By targeting specific pathways involved in ROS generation and antioxidant defense mechanisms, novel therapeutic strategies may help alleviate oxidative stress and attenuate CKD progression. Further research is needed to establish the complex interplay between oxidative stress and CKD pathophysiology and therefore, to develop novel effective therapeutic interventions.

## 4. Fibrosis

The intricacy of renal fibrogenesis, a pivotal process in CKD, is characterized by the accumulation of fibrous tissue in the kidneys. Renal fibrogenesis involves a series of intricate cellular and molecular events orchestrated by various signaling pathways, ultimately leading to a progressive decline in kidney function. Renal fibrogenesis progresses through distinct phases, including priming, activation, execution, and progression. The initial phase involves nonresolving inflammation, which primes the kidney for fibrosis and activates matrix-producing fibroblasts [54]. Various cellular mechanisms contribute to renal fibrogenesis. These multifaceted mechanisms underlying renal fibrosis are presented in Table 1 [55].

Fibroblasts play a central role, transitioning into myofibroblasts and producing extracellular matrix (ECM) proteins. Epithelial–mesenchymal transition (EMT) and endothelial-to-mesenchymal transition (EndoMT) also contribute to fibrotic tissue formation. These cellular processes are regulated by signaling pathways activated in response to injury and inflammation [54].

Indeed, it is worth noting that there are several potential sources of myofibroblasts, including the possibility of transdifferentiation of various cell types present in the kidneys. One of these potential sources is interstitial fibroblasts, which are cells crucial for maintaining extracellular matrix homeostasis in the kidneys. In the context of kidney injury, interstitial fibroblasts can become activated and transform into myofibroblasts, which are pivotal for the fibrosis process. Another potential source of myofibroblasts is vascular pericytes, which are cells surrounding blood vessels. It follows that vascular cells may have the ability to differentiate into myofibroblasts in response to kidney injury [56]. The renal microenvironment plays a critical role in modulating fibrogenic signaling. Chronic hypoxia, oxidative stress, and inflammation contribute to fibrosis progression by inducing cellular responses that promote ECM synthesis and deposition. These microenvironmental factors create a favorable milieu for fibrosis development and exacerbation of kidney damage [54].

An important role is also played by the antioxidant enzyme GPX3. The deficiency of GPX3 leads to the formation of a microenvironment with increased oxidative stress. In turn, NOX4 is an enzyme responsible for the production of ROS in fibroblast activation. It has been shown that the lack of GPX3 leads to the induction of NOX4 through the generation of oxidative stress in the surrounding microenvironment, which in turn promotes further fibroblast activation [57]. An activated form of interstitial fibroblast—the α-smooth muscle actin-positive myofibroblast—is widely recognized as the major type of matrix-producing cell in the fibrotic kidney. Studies show that tubular epithelial cells only undergo a partial EMT during renal fibrosis, so complete phenotypic conversion of tubular epithelial cells to a myofibroblast phenotype is extremely rare, if occurring at all. Nevertheless, this partial EMT is sufficient to induce tubular function impairment, triggering cell cycle arrest and promoting the release of critical fibrogenic cytokines. One of the functional consequences of partial EMT is the induction of arrest in the G2 phase of the cell cycle, which compromises the potential of tubular epithelial cells to repair and regenerate. Renal tubular epithelial cells undergo a partial EMT after injury, which impairs tubular repair and regeneration, induces cell cycle arrest, and drives interstitial fibroblast activation [58].

There are various factors influencing kidney fibrosis such as:

1. Genetic factors: variations in genes relevant to immune response, injury response, drug metabolism, and fibrosis could significantly impact for instance graft prognosis. MicroRNA (miRNA) also plays a contributory role in renal fibrosis, especially in the context of CKD and diabetes. Some miRNAs act as profibrotic factors, contributing to excessive collagen deposition and tissue fibrosis, while others act as antifibrotic factors, protecting both the kidneys and the heart [59]. Epigenetic alterations, particularly DNA methylation and histone modification, are pivotal in driving kidney fibrosis progression. DNA methylation, involving the addition of methyl groups to specific CpG sites, influences gene expression patterns implicated in renal fibrosis and the progression of CKD. Histone acetylation and deacetylation mechanisms play crucial roles in modulating gene expression linked to fibrosis. Histone acetylation facilitates chromatin opening, making DNA more accessible for transcription, while histone deacetylation represses gene expression. Both processes regulate key fibrotic pathways, including EMT, myofibroblast activation, inflammation, and profibrotic factor secretion [60];
2. Viral infections: certain viral infections, particularly polyomavirus-associated nephropathy (PVAN) and cytomegalovirus (CMV), have been associated with more severe fibrosis in renal allografts. PVAN, if left untreated, can lead to rapid accumulation of fibrosis;
3. Various other factors, such as infections caused by human herpesviruses and Epstein–Barr virus, as well as the nephrotoxic effects of immunosuppressive drugs, contribute to fibrosis progression [61].

These factors collectively contribute to the complex interplay of immune responses, injury mechanisms, and fibrotic processes in renal transplantation, highlighting the multifactorial nature of fibrosis progression [59].

A paradoxical role of fibrosis is observed in the context of kidney diseases. Fibrosis can serve two opposing functions: it can be both a reparative mechanism and a factor leading to the progression of kidney diseases. Fibrosis can be perceived as a compensatory mechanism that aids in the repair and stabilization of the kidney structure. In response to damage to the renal parenchyma, myofibroblasts may activate and begin to produce excess connective tissue, leading to scar formation and filling of defects in the kidney tissue. This process may help maintain the structural integrity of the kidneys by preventing the spread of damage and stabilizing the parenchyma. However, despite its positive role in tissue repair, fibrosis leads to the progression of kidney diseases over a longer period. Excessive production of connective tissue and scar formation may lead to a decrease in the number of healthy nephrons and disruption of blood flow through the kidney. As a result, kidney function may worsen, leading to progressive loss of its ability to perform its basic functions [62]. This process is depicted as a pivotal element in the progression of CKD.

It is worth mentioning that articles addressing therapies aimed at halting or even reversing the progression of CKD highlight the limitations of currently available therapeutic methods. Therefore, increased attention is being focused on therapies aimed at reducing renal fibrosis. An example of such therapy is targeting the reduction in (TGF-β expression. TGF-β plays a crucial role in the pathogenesis of renal fibrosis by stimulating mesangial cell proliferation, and extracellular matrix production, and inducing EMT of tubular epithelial cells into mesenchymal cells, contributing to excessive collagen and other fibrotic protein production. However, most studies are conducted in the late stages of the disease, when the fibrotic process is already advanced. Therefore, even if the therapy effectively inhibits TGF-β, it may be too late to reverse existing fibrosis [63].

## 5. Treatment and Novel Therapies

### 5.1. Glucagon-like Peptide-1 (GLP-1) Agonists

GLP-1 agonists have emerged as promising pharmacological agents for addressing the multifaceted pathophysiology of CKD. These agents mimic the action of GLP-1, an incretin hormone produced in the intestine in response to food intake [64]. By activating GLP-1 receptors, which are abundant in various tissues including the pancreas, gastrointestinal tract, cardiovascular system, central nervous system, and kidneys, GLP-1 agonists exert their effects. For example, they enhance glucose-dependent insulin secretion, inhibit glucagon release, delay gastric emptying, suppress appetite, promote vasodilation, and confer cardioprotection [65,66]. Additionally, GLP-1 agonists may improve renal function and attenuate renal fibrosis. This comprehensive mechanism of action offers new avenues for managing metabolic disorders and holds promise for improving outcomes in patients with CKD [66,67].

One of the critical complications of CKD is the accumulation of uremic toxins due to impaired renal clearance, leading to systemic inflammation and cardiovascular complications. Muskiet et al. (2017) provided an extensive review of the physiological and pharmacological effects of GLP-1 on the kidney, highlighting its role in improving renal function and reducing uremic toxin burden in CKD. Experimental and clinical evidence supports the beneficial effects of GLP-1 receptor agonists on kidney outcomes in diabetes and CKD [68]. The mechanism of action of GLP-1 agonists involves several pathways in the kidneys. Activation of GLP-1 receptors on renal tubular cells enhances sodium excretion and diuresis, leading to improved fluid balance and blood pressure regulation. GLP-1 receptor activation also promotes renal vasodilation by stimulating nitric oxide production and inhibiting the renin–angiotensin–aldosterone system (RAAS), thereby improving renal blood flow and reducing intraglomerular pressure [69]. Oxidative stress is another hallmark of CKD, resulting from an imbalance between ROS production and antioxidant defenses. Winiarska et al. (2021) conducted a comprehensive review discussing the role of inflammation and oxidative stress in diabetic kidney disease (DKD) and the potential therapeutic targets of SGLT2 inhibitors and GLP-1 receptor agonists in mitigating these pathophysiological processes. Their review synthesized evidence from preclinical and clinical studies, highlighting the antioxidative and anti-inflammatory properties of GLP-1 receptor agonists in DKD. They elucidated how GLP-1 receptor agonists may mitigate oxidative stress by enhancing antioxidant defenses and reducing ROS production in the diabetic kidney microenvironment. These findings provide valuable insights into the mechanisms underlying the renoprotective effects of GLP-1 agonists and their potential implications for managing DKD [70]. Progressive renal fibrosis, characterized by excessive extracellular matrix deposition, is a key determinant of CKD progression to end-stage renal disease. Li YK et al. (2018) investigated the effects of liraglutide, a GLP-1 receptor agonist, on renal fibrosis in a preclinical model of chronic kidney disease. Their study demonstrated that liraglutide treatment attenuated renal fibrosis by inhibiting TGF-β signaling, reducing inflammation, and suppressing fibroblast activation. Specifically, liraglutide administration led to decreased expression of TGF-β, a central mediator of fibrogenesis, and its downstream profibrotic targets such as alpha-smooth muscle actin (α-SMA) and collagen type I. Moreover, liraglutide treatment was associated with reduced infiltration of inflammatory cells and decreased production of pro-inflammatory cytokines in the kidney microenvironment. These findings provide experimental evidence supporting the therapeutic potential of liraglutide in mitigating renal fibrosis in chronic kidney disease [71]. In accordance with the KDIGO 2024 Clinical Practice Guideline for the Evaluation and Management of Chronic Kidney Disease, specific recommendations have been made regarding the use of GLP-1 receptor agonists in the management of type 2 diabetes (T2D) and CKD. Recommendations suggest that in adults with T2D and CKD who have not achieved individualized glycemic targets despite the use of metformin and SGLT2 inhibitor treatment, or who are unable to use those medications, a long-acting GLP-1 receptor agonist is recommended. In addition, they emphasized the importance of prioritizing GLP-1 RA with documented cardiovascular benefits when choosing treatment [3,72].

GLP-1-based therapies hold significant promise in the management of CKD by addressing multiple aspects of the disease simultaneously. While further clinical studies are needed to establish their long-term efficacy and safety profile in CKD patients, the multifaceted pharmacological properties of GLP-1 analogs offer new avenues for comprehensive disease management and improved patient outcomes [73,74,75].

### 5.2. Sodium-Glucose Cotransporter Protein 2 (SGLT2) Inhibitors

**Figure 3:**
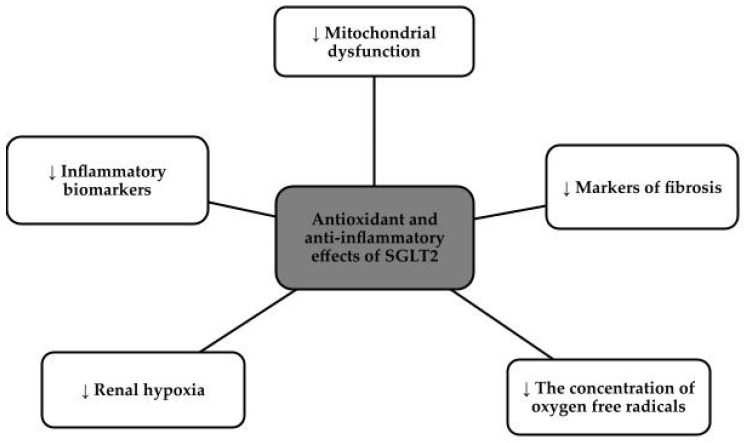
Antioxidant and anti-inflammatory effects of SGLT2 [82].

SGLT2 inhibitors, otherwise known as gliflozins, i.e., canagliflozin, dapagliflozin, empagliflozin, and ertugliflozin, for example, initially made a name for themselves as hypoglycemic drugs, but currently dominate as cardiovascular and nephroprotective drugs [76,77]. These drugs came to prominence in 2012 when both the Food and Drug Administration in the United States (FDA) and European Medicines Agency (EMA) approved their use [78]. The approach to this group of drugs changed after the publication of three major studies that showed a clear reduction in complications and events both cardiovascular and reducing the risk of renal events, namely the Canagliflozin Cardiovascular Assessment Study (CANVAS) program, the Empagliflozin Cardiovascular Outcome Event Trial (EMPA-REG OUTCOME), and the Dapagliflozin Effect on Cardiovascular Events-Thrombolysis in Myocardial Infarction 58 (DECLARE-TIMI 58) trial [76,79]. SGLT2 inhibitors, via proximal tubules, prevent the absorption of both glucose and sodium chloride. Reabsorption of Na+ in the renal tubules, decreases intraglomerular pressure which directly translates into decreased glomerular hyperfiltration, reduced oxidative stress and slowed progression of chronic kidney disease and nephropathy [80,81]. This group of drugs has potent anti-inflammatory effects, due to the down-regulation of the nucleotide-binding domain, or NLRP3. This protein is responsible for vascular endothelial damage and the progression of the fibrosis process, so lowering its levels has a beneficial effect on inhibiting the progression of chronic kidney disease. In addition, SGLT2 inhibitors reduce the concentration of pro-inflammatory factors and contribute to the beneficial phenomenon of autophagy. SGLT2 inhibitors reduce hyperglycemia, hypertension, albuminuria, fibrosis, hypoxia, obesity, lipotoxicity and inflammation [80]. Figure 3 shows the effects of SGLT2 inhibitors on inflammation and oxidative stress [82].

A study in mice with type 2 diabetes showed that SGLT2 inhibitors prevented renal tubular fibrosis [80]. Other studies show that these drugs can reduce abnormal cell remodeling by decreasing endothelial markers. An increase in the eNOS enzyme has been shown to inhibit pathological remodeling in diabetic kidney disease by restoring proper NO utilization and having a protective effect on both the renal endothelium and the cardiovascular system [83]. One of the main pro-fibrotic factors is growth factor 1 (TGF-1), which has potent profibrotic effects through the deposition of the extramesenchymal matrix, resulting in interstitial fibrosis and glomerular sclerosis. In this study, conducted in Ojima, checked the effect of empagliflozin on the kidneys of rats with type 2 diabetes mellitus. After one month of using the drug, inhibition of oxidative stress by reducing the expression of AGE (Advanced Glycation End-products) and RAGE (Receptor for Advanced Glycation End-products) proteins was discovered. Thus, it can be concluded that empagliflozin protects renal tubules from fibrosis, has an anti-inflammatory effect and, by reducing oxidative stress, has a protective effect on renal tissues [81]. SGLT2 inhibitors additionally have a protective effect on the kidneys by preserving oxygenation of primarily the renal cortex, which is crucial for normal renal function [84]. SGLT2 inhibitors cause an increase in diuresis including glucosuria and natriuresis, and a decrease in hyperfiltration, oxidative stress, inflammation and fibrosis [78]. In addition, it was proven that SGLT2 inhibitors showed a reduction in severe hyperkalemia, which also had a protective effect on the kidneys [85]. It is worth noting that SGLT2 inhibitors should be used both in the primary prevention of CKD in a population at increased risk of diabetes and regardless of the cause [86]. The DAPA-CKD trial, which was conducted in 2020 and prematurely terminated, involved more than 4,000,000 patients. Despite its early termination, it showed a positive effect of one of the SGLT2 inhibitors, dapagliflozin, regardless of the presence or absence of T2DM. It showed an almost 27% reduction in renal endpoints and a reduction in the frequency of cardiovascular events. Therefore, SGLT2 inhibitors also have their nephroprotective and cardioprotective effects in patients without T2DM [78].

### 5.3. Finerenone

Finerenone is a new non-steroidal mineralocorticosteroid receptor antagonist (MRA) that has an indication for use in patients with chronic kidney disease and coexisting albuminuria associated with type 2 diabetes, with administration of maximum well-acceptable doses of drugs such as angiotensin-converting enzyme inhibitors (ACEIs) and angiotensin II receptor blockers (ARBs) [87,88]. The drug was approved by the FDA in July 2021, by the EMA in February 2022 and by the Drug Controller General from India (DCGI) in April 2022, with an indication in patients with type 2 diabetes and chronic kidney disease [89,90]. In addition to inhibiting the progression of chronic kidney disease, it has also been shown to have beneficial effects on the cardiovascular system, thereby reducing the rate of complications in this group of patients [87]. Thus, a multicenter, randomized, double-blinded study—Finerenone in Reducing Kidney Failure and Disease Progression in Diabetic Kidney Disease (FIDELIO-DKD)—was performed in which more than 5500 people with type 2 diabetes, CKD, albuminuria and falling between GFR 25–75 mL/min/1.73 m^2^ participated [91]. It was shown, after 2.6 years of follow-up, that finerenone compared to placebo significantly reduced the incidence of renal complications, including death, in patients with the entire spectrum of CKD [91,92]. As for the mechanism of action, it is worth noting that there are substances that cause increased mineralocorticosteroid (MR) activation such as cortisol, progesterone, hypernatremia, increased glucose, aldosterone ligands or RAC1 non-ligands. Thus, excessive MR activation should be inhibited, thus reducing inflammation and fibrosis, resulting in a reduction in the progression of chronic kidney disease [87]. In studies of CKD, it has been shown that the presence of aldosterone caused a number of adverse effects, such as increasing the concentration of reactive oxygen species, increasing collagen synthesis, which resulted in the formation of renal cortical fibrosis and increased inflammation. There was concomitant damage to one of the elements of the glomerular filtration barrier, namely podocytes. In vascular endothelial cells, aldosterone caused significant dysfunction of this endothelium and oxidative destruction of vessels [93]. Finerenone has distinct features that differentiate it from other drugs in the MRA group. Where the steroidal MRAs we are familiar with, such as eplerenone or spironolactone, have partial agonism in the recruitment of cofactors, the non-steroidal MRA finerenone is a passive antagonist in the inhibition of said cofactors. Therefore, finerenone has an advantage over the other drugs in this group because it is both potent and more selective to the MRAs we know [89]. Thanks to the fact that finerenone has a balanced distribution between the kidneys and the heart, and not, like the other steroidal MRAs, only in the kidneys, the use of this drug does not cause a risk of hyperkalemia, as confirmed by Phase I and Phase II clinical trials [87,93]. Finerenone has no active metabolites, has negligible urinary and biliary excretion, and has an average half-life of 2–3 h [89,90,94]. The drug is completely absorbable and undergoes first-pass metabolism, due to which its bioavailability is more than 43% [94]. Thanks to its potency and selectivity, finerenone does not cause side effects such as gynecomastia or the aforementioned hyperkalemia [89]. The recommended daily dose is 10–20 mg ×1 per day, depending on GFR and blood potassium levels, with all dose reductions requiring every 4 weeks monitoring of the aforementioned blood parameters [87]. Finerenone at a dose of 20 mg, in studies, correlated with lower blood potassium concentrations than with finereon at a dose of 10 mg [95]. As for the effect on blood pressure, it is small, while the drug makes up for it by having a strong proteinuria-reducing effect and showing great renal protection with a moderate risk of hyperkalemia [96]. Numerous studies, therefore, provide evidence that this drug has both anti-inflammatory and antifibrotic effects, so that tissue remodeling is not as strong and we obtain an antiproliferative effect to both the urinary and cardiovascular systems [93,94]. The use of non-steroidal MRAs, according to studies, plays a key role in reducing fibrosis and sclerosis not only in the glomeruli but also in the vascular endothelium and renal interstitium [97]. For now, we have to wait for papers and studies raising the topic of how to combine, and whether to combine at all, in patients the non-steroidal MRA—finerenone and the previously described SGLT2 [93]. This is certainly a very interesting topic and very important in the context of inhibiting the progression of chronic kidney disease.

### 5.4. Canakinumab

Canakinumab, a monoclonal antibody targeting interleukin-1 beta (IL-1β), has emerged as a promising therapeutic approach for addressing the inflammatory component of chronic kidney disease (CKD). By specifically binding to IL-1β and inhibiting its activity, Canakinumab effectively dampens the inflammatory cascade, thereby attenuating renal injury and preserving kidney function [98,99]. Emerging evidence suggests a multifaceted role for Canakinumab in mitigating inflammation-associated complications in CKD patients [100]. The CANTOS (Canakinumab Anti-inflammatory Thrombosis Outcomes Study) trial, conducted by Ridker et al., represents a landmark study evaluating the cardiovascular effects of Canakinumab in patients with a history of myocardial infarction and elevated high-sensitivity CRP levels. This randomized, double-blind, placebo-controlled trial included over 10,000 participants and demonstrated that Canakinumab significantly reduced the risk of major adverse cardiovascular events, including myocardial infarction, stroke, and cardiovascular death, independent of lipid lowering. Importantly, the trial provided evidence for the role of inflammation in atherosclerosis and highlighted Canakinumab’s potential as an anti-inflammatory therapy for cardiovascular disease [101,102]. Clinical studies have also shown the efficacy of Canakinumab in reducing markers of systemic inflammation, such as C-reactive protein (CRP) and interleukin-6 (IL-6), and improving renal function in CKD populations. Ridker et al. (2017) reported that Canakinumab treatment led to a significant reduction in CRP levels, suggesting its potent anti-inflammatory effects [103,104]. Furthermore, preclinical models have provided insights into the molecular mechanisms underlying Canakinumab’s renoprotective effects. Ridker et al. (2020) investigated interleukin-6 signaling and anti-interleukin-6 therapeutics in cardiovascular disease, shedding light on the broader implications of targeting IL-6 in inflammatory conditions [105]. Canakinumab’s therapeutic potential extends beyond its anti-inflammatory effects to encompass its cardiovascular benefits in CKD. Cardiovascular disease represents a leading cause of morbidity and mortality in CKD patients, with inflammation serving as a key driver of atherosclerosis and cardiovascular events. Canakinumab’s ability to mitigate systemic inflammation may confer cardiovascular protection and reduce the risk of adverse cardiovascular outcomes in CKD patients [106,107]. In the context of CKD management, Canakinumab offers a novel therapeutic strategy aimed at targeting inflammation-driven pathways implicated in disease progression. While the KDIGO guidelines primarily focus on the management of CKD complications, recent updates have acknowledged the potential role of anti-inflammatory agents, such as Canakinumab, in improving renal outcomes [3]. Notably, Canakinumab (Ilaris) is approved by the FDA to treat adult-onset Still’s disease (AOSD) and systemic juvenile idiopathic arthritis. Future research endeavors, including clinical trials and mechanistic studies, are warranted to further elucidate Canakinumab’s therapeutic role in CKD and optimize its clinical utility [108,109]. This addition highlights Canakinumab’s FDA approval for specific indications and emphasizes its potential as an anti-inflammatory agent in the management of CKD, as well as its relevance in the context of KDIGO guidelines [3,108,109].

### 5.5. Pirfenidone

**Figure 4:**
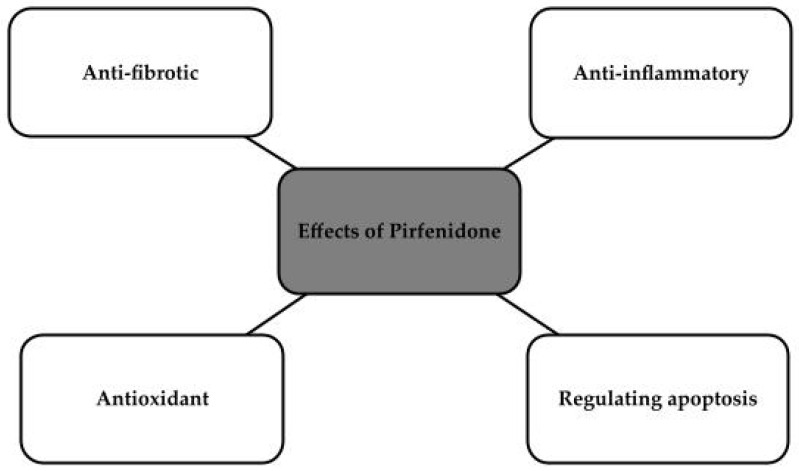
Effects of Pirfenidone [112,114].

Pirfenidone, or 5-methyl-1-phenyl-2-1H-pyridin-2-one, is a non-peptide molecule with a molecular weight of 185 g/mol and high bioavailability [110,111,112]. It has been approved by the FDA, by Japan and by the European Union. It has been approved for the treatment of idiopathic pulmonary fibrosis. The drug’s mechanism of action has proven to be very interesting, and its anti-fibrotic effects in chronic heart disease and kidney disease have been considered. It is also used to treat skin ulcers or wounds [111]. The drug is an inhibitor of transforming growth factor-b, which has antioxidant effects that largely reduce glomerular sclerosis, which could be used in chronic kidney disease [113]. It is also said to have several mechanisms that could be used to inhibit the progression of chronic diseases of both heart and kidney, as shown in Figure 4 [112,114].

The drug is mainly metabolized in the liver, by CYP1A2, and will therefore be contraindicated in patients with liver disease or liver failure [111].

Pirfenidone is recommended to be administered orally, thus minimizing side effects such as nausea and vomiting. Other studied effects include the appearance of skin rashes or hypersensitivity to light [111].

In experimental studies, on models that had kidney damage such as hypertension, diabetic nephropathy or partial nephrectomy, pirfenidone supply resulted in reduced proteinuria, lower GFR, reduced mesangial proliferation, reduced macrophage infiltration and renal fibrosis. Following the experimental studies, a randomized, placebo-controlled, double-blind study was conducted, where patients with diabetic nephropathy showed an increase in glomerular filtration rate after one year of the drug compared to placebo. Because of this promising study, the drug is also being considered for use in reducing damage to the structure of the kidney caused by inadequate oxygen supply to its cells [110].

### 5.6. Sacubitril/Valsartan

Entresto combines sacubitril, a neprilysin inhibitor, with valsartan (ARB) to target multiple pathways implicated in CKD pathogenesis. Neprilysin inhibition enhances the bioavailability of natriuretic peptides, pivotal regulators of renal function and fluid balance. Natriuretic peptides exert vasodilatory, natriuretic, and diuretic effects, promoting renal blood flow, sodium excretion, and urine output. Moreover, they antagonize the actions of the RAAS, thereby counteracting vasoconstriction, sodium retention, and fibrosis in the kidneys [115,116]. Concurrently, ARB-mediated blockade of angiotensin II further complements the renoprotective effects of sacubitril. Angiotensin II, a potent vasoconstrictor and pro-inflammatory mediator, contributes to renal injury by promoting inflammation, and fibrosis. By inhibiting the angiotensin II type 1 (AT1) receptor, valsartan attenuates these deleterious effects, leading to improved renal hemodynamics, reduced inflammation, and mitigated fibrosis. Additionally, ARB therapy may decrease proteinuria, a key marker of renal damage and progression in CKD, by preserving glomerular integrity and reducing podocyte injury [117,118]. Numerous studies support the efficacy of Sacubitril/valsartan in CKD management. One pivotal trial, the Prospective Comparison of ARNI with ACEI to Determine Impact on Global Mortality and Morbidity in Heart Failure (PARADIGM-HF) trial, demonstrated its superiority over enalapril in reducing the risk of cardiovascular death or heart failure hospitalization in patients with heart failure with reduced ejection fraction (HFrEF). This landmark trial enrolled over 8000 patients with HFrEF and showed that sacubitril/valsartan significantly reduced the primary composite endpoint of cardiovascular death or heart failure hospitalization by 20% compared to enalapril. Importantly, sacubitril/valsartan also led to significant reductions in renal function decline and adverse renal outcomes compared to enalapril, highlighting its potential renoprotective effects in addition to its cardiovascular benefits [116,119]. Subsequent analyses of the PARADIGM-HF trial data have provided further insights into the renal effects of sacubitril/valsartan. These analyses revealed that sacubitril/valsartan treatment was associated with a slower decline in eGFR over time compared to enalapril, suggesting a potential renal protective effect beyond its hemodynamic benefits. Additionally, sacubitril/valsartan was associated with a lower incidence of adverse renal outcomes, including the need for renal replacement therapy or doubling of serum creatinine, compared to enalapril-treated patients. These findings underscore the importance of sacubitril/valsartan as a promising therapeutic option for patients with heart failure and concomitant CKD, offering both cardiovascular and renal benefits [116,119,120]. Uremic toxins contribute significantly to systemic inflammation and cardiovascular complications. By enhancing natriuretic peptide activity and modulating renal hemodynamics, sacubitril/valsartan reduces the burden of uremic toxins, potentially improving clinical outcomes for CKD patients [121]. Sacubitril/valsartan’s dual mechanism of action extends beyond hemodynamic modulation to include antioxidant properties. By inhibiting the RAAS and enhancing natriuretic peptide signaling, Entresto may mitigate oxidative stress, thereby preserving renal function and attenuating disease progression [122]. Renal fibrosis, characterized by excessive extracellular matrix deposition, further exacerbates CKD pathology. Sacubitril/valsartan shows promise in attenuating renal fibrosis through its comprehensive approach. Neprilysin inhibition counters profibrotic pathways and promotes vasodilation, while ARB-mediated angiotensin II blockade mitigates inflammation and fibroblast activation. This synergistic action targets multiple pathways involved in renal fibrosis, potentially slowing disease progression and preserving renal function [123]. Sacubitril/valsartan (Entresto) represents a paradigm shift in CKD management, offering a holistic approach to address uremic toxins, oxidative stress, and renal fibrosis. As further research elucidates its full therapeutic potential, Entresto stands poised to redefine the treatment landscape for CKD, offering hope for improved outcomes and quality of life for patients worldwide [124,125].

## 6. Conclusions

The kidneys play a crucial role in filtering waste products and excess fluids from the blood, regulating electrolyte balance, and producing hormones that help control blood pressure and red blood cell production. In conclusion, CKD is a multifaceted condition with far-reaching implications for individuals and healthcare systems worldwide. By understanding its mechanisms, we could work towards early detection, effective management, and improved outcomes for those affected by this chronic disease.

Patients in the course of CKD experience excessive accumulation of UTs, which have adverse effects on the body and the progression of the disease. UTs can be divided based on their physicochemical properties into three main groups: small water-soluble compounds (<500 Da), mid-sized molecules (>500 Da) and protein-bound compounds (<500 Da). The accumulation of UTs in the body, especially those bound to proteins, leads to increased oxidative stress, endothelial dysfunction, the development of atherosclerosis, cardiovascular events and even renal fibrosis.

Oxidative stress plays a critical role in the pathogenesis of CKD, contributing to renal injury, systemic complications, and disease progression. Understanding these mechanisms is crucial for developing targeted therapeutic interventions to mitigate renal injury and improve patient outcomes. By addressing inflammation and oxidative stress, we may unlock new avenues for preventing and treating CKD and its associated complications.

The insights gained from many studies offer potential therapeutic targets for the treatment of renal fibrosis. Inhibition of EMT-related pathways or targeting of developmental signaling pathways holds promise for attenuating fibrosis and preserving kidney function in chronic kidney disease. Also, genetic discoveries in the field of kidney disease emphasize the importance of epigenetic modifications as potential targets for therapeutic interventions aimed at mitigating the progression of renal fibrosis. Furthermore, it is worth emphasizing the significance of tubular regeneration as a key mechanism in limiting the progression of fibrosis and chronic kidney diseases. The kidney’s ability to regenerate damaged tubules may play a crucial role in preventing fibrosis development and maintaining renal function. Therefore, focusing on supporting the tubular regeneration process may represent a promising therapeutic strategy in treating kidney diseases, allowing for the preservation of renal function and the delay of disease progression.

There are many groups of drugs that are undergoing numerous experimental and clinical studies to determine their effect on halting the progression of chronic kidney disease. The latest of these raise the subject of modernizing oxidative stress and reducing the process of vascularization. In the present study, we focused on the impact of well-known and used drug groups such as SGLT2 inhibitors, GLP-1 analogs and Sacubitril/Valsartan but also raised the topic of newer drugs such as finerenone, canacinumab and pirfenidone. The topic is not fully researched and developed, but offers a light at the tunnel for new therapeutic possibilities.

## Figures and Tables

**Table 1:** The mechanisms responsible for renal fibrosis [55].

Mechanism	Significance
TGF-B1 and Wnt/ B-catenin pathways	Crucial for the development of renal fibrosis, leading to the expression of profibrotic genes and the transition of fibroblasts into myofibroblasts.
Various cell death pathways	Apoptosis, necroptosis, and ferroptosis. A significant role in inflammatory and pro-fibrotic reactions, contributing to renal damage.
Regulation by hypoxia-inducible factor (HIF)	HIF influences the expression of genes related to the inflammatory response, angiogenesis, and metabolism regulation.
Mitochondrial dysfunction	Disturbances in mitochondrial homeostasis contribute to renal damage through processes such as mitochondrial fusion, mitophagy, and mitochondrial biogenesis.
Cell cycle arrest in the G2/M phase	Activation of profibrotic factors.
Immune response	Both innate and adaptive immune reactions.

## References

[1] Charles C., Ferris A.H. (2020). Chronic Kidney Disease. Prim. Care.

[2] Ammirati A.L. (2020). Chronic Kidney Disease. Rev. Assoc. Med. Bras..

[3] Kidney Disease: Improving Global Outcomes (KDIGO) CKD Work Group (2024). KDIGO 2024 Clinical Practice Guideline for the Evaluation and Management of Chronic Kidney Disease. Kidney Int..

[4] Vallianou N.G., Mitesh S., Gkogkou A., Geladari E. (2019). Chronic Kidney Disease and Cardiovascular Disease: Is there Any Relationship?. Curr. Cardiol. Rev..

[5] Jagieła J., Bartnicki P., Rysz J. (2020). Selected cardiovascular risk factors in early stages of chronic kidney disease. Int. Urol. Nephrol..

[6] Gaitonde D.Y., Cook D.L., Rivera I.M. (2017). Chronic Kidney Disease: Detection and Evaluation. Am. Fam. Physician.

[7] Wu C.L., Tarng D.C. (2020). Targeting Uremic Toxins to Prevent Peripheral Vascular Complications in Chronic Kidney Disease. Toxins.

[8] Lim Y.J., Sidor N.A., Tonial N.C., Che A., Urquhart B.L. (2021). Uremic Toxins in the Progression of Chronic Kidney Disease and Cardiovascular Disease: Mechanisms and Therapeutic Targets. Toxins.

[9] Rosner M.H., Reis T., Husain-Syed F., Vanholder R., Hutchison C., Stenvinkel P., Blankestijn P.J., Cozzolino M., Juillard L., Kashani K. (2021). Classification of Uremic Toxins and Their Role in Kidney Failure. Clin. J. Am. Soc. Nephrol..

[10] Fujii H., Goto S., Fukagawa M. (2018). Role of Uremic Toxins for Kidney, Cardiovascular, and Bone Dysfunction. Toxins.

[11] André C., Bodeau S., Kamel S., Bennis Y., Caillard P. (2023). The AKI-to-CKD Transition: The Role of Uremic Toxins. Int. J. Mol. Sci..

[12] Wolley M.J., Hutchison C.A. (2018). Large uremic toxins: An unsolved problem in end-stage kidney disease. Nephrol. Dial. Transplant..

[13] Addi T., Dou L., Burtey S. (2018). Tryptophan-Derived Uremic Toxins and Thrombosis in Chronic Kidney Disease. Toxins.

[14] Hung S.C., Kuo K.L., Wu C.C., Tarng D.C. (2017). Indoxyl Sulfate: A Novel Cardiovascular Risk Factor in Chronic Kidney Disease. J. Am. Heart Assoc..

[15] Vanholder R., Schepers E., Pletinck A., Nagler E.V., Glorieux G. (2014). The uremic toxicity of indoxyl sulfate and p-cresyl sulfate: A systematic review. J. Am. Soc. Nephrol..

[16] Lauriola M., Farré R., Evenepoel P., Overbeek S.A., Meijers B. (2023). Food-Derived Uremic Toxins in Chronic Kidney Disease. Toxins.

[17] Liabeuf S., Laville S.M., Glorieux G., Cheddani L., Brazier F., Titeca Beauport D., Valholder R., Choukroun G., Massy Z.A. (2020). Difference in Profiles of the Gut-Derived Tryptophan Metabolite Indole Acetic Acid between Transplanted and Non-Transplanted Patients with Chronic Kidney Disease. Int. J. Mol. Sci..

[18] Dou L., Sallée M., Cerini C., Poitevin S., Gondouin B., Jourde-Chiche N., Fallague K., Brunet P., Calaf R., Dussol B. (2015). The cardiovascular effect of the uremic solute indole-3 acetic acid. J. Am. Soc. Nephrol..

[19] Long Y., Nie J. (2016). Homocysteine in Renal Injury. Kidney Dis..

[20] Cohen E., Margalit I., Shochat T., Goldberg E., Krause I. (2019). The relationship between the concentration of plasma homocysteine and chronic kidney disease: A cross sectional study of a large cohort. J. Nephrol..

[21] Perna A.F., Ingrosso D. (2019). Homocysteine and chronic kidney disease: An ongoing narrative. J. Nephrol..

[22] Ostrakhovitch E.A., Tabibzadeh S. (2015). Homocysteine in Chronic Kidney Disease. Adv. Clin. Chem..

[23] Graboski A.L., Redinbo M.R. (2020). Gut-Derived Protein-Bound Uremic Toxins. Toxins.

[24] Lu P.H., Yu M.C., Wei M.J., Kuo K.L. (2021). The Therapeutic Strategies for Uremic Toxins Control in Chronic Kidney Disease. Toxins.

[25] Nangaku M., Mimura I., Yamaguchi J., Higashijima Y., Wada T., Tanaka T. (2015). Role of uremic toxins in erythropoiesis-stimulating agent resistance in chronic kidney disease and dialysis patients. J. Ren. Nutr..

[26] Yiang G.T., Su W.L., Zheng C.M., Liao M.T., Cheng T.H., Lu C.L., Lu K.C. (2023). The influence of uremic toxins on low bone turnover disease in chronic kidney disease. Tzu Chi Med. J..

[27] Black A.P., Cardozo L.F., Mafra D. (2015). Effects of Uremic Toxins from the Gut Microbiota on Bone: A Brief Look at Chronic Kidney Disease. Ther. Apher. Dial..

[28] Roumeliotis S., Liakopoulos V., Dounousi E., Mark P.B. (2023). Oxidative Stress in End-Stage Renal Disease: Pathophysiology and Potential Interventions. Oxidative Med. Cell. Longev..

[29] Irazabal M.V., Torres V.E. (2020). Reactive Oxygen Species and Redox Signaling in Chronic Kidney Disease. Cells.

[30] Jiang W., Zhou Y., Chen S., Liu S. (2023). Impact of Chronic Kidney Disease on Outcomes of Percutaneous Coronary Intervention in Patients with Diabetes Mellitus: A Systematic Review and Meta-Analysis. Tex. Heart Inst. J..

[31] Piko N., Bevc S., Hojs R., Ekart R. (2023). The Role of Oxidative Stress in Kidney Injury. Antioxidants.

[32] Kadatane S.P., Satariano M., Massey M., Mongan K., Raina R. (2023). The Role of Inflammation in CKD. Cells.

[33] Machowska A., Carrero J.J., Lindholm B., Stenvinkel P. (2016). Therapeutics targeting persistent inflammation in chronic kidney disease. Transl. Res..

[34] Fu Y., Xiang Y., Li H., Chen A., Dong Z. (2022). Inflammation in kidney repair: Mechanism and therapeutic potential. Pharmacol. Ther..

[35] Morgan M.J., Liu Z.G. (2011). Crosstalk of reactive oxygen species and NF-κB signaling. Cell Res..

[36] Nezu M., Suzuki N., Yamamoto M. (2017). Targeting the KEAP1-NRF2 System to Prevent Kidney Disease Progression. Am. J. Nephrol..

[37] Saito H. (2013). Toxico-pharmacological perspective of the Nrf2-Keap1 defense system against oxidative stress in kidney diseases. Biochem. Pharmacol..

[38] Zuk A., Bonventre J.V. (2016). Acute Kidney Injury. Annu. Rev. Med..

[39] Sato Y., Takahashi M., Yanagita M. (2020). Pathophysiology of AKI to CKD progression. Semin. Nephrol..

[40] Rossi M., Delbauve S., Wespes E., Roumeguère T., Leo O., Flamand V., Le Moine A., Hougardy J.M. (2018). Dual effect of hemin on renal ischemia-reperfusion injury. Biochem. Biophys. Res. Commun..

[41] Tejchman K., Kotfis K., Sieńko J. (2021). Biomarkers and Mechanisms of Oxidative Stress-Last 20 Years of Research with an Emphasis on Kidney Damage and Renal Transplantation. Int. J. Mol. Sci..

[42] Fontecha-Barriuso M., Lopez-Diaz A.M., Guerrero-Mauvecin J., Miguel V., Ramos A.M., Sanchez-Niño M.D., Ruiz-Ortega M., Ortiz A., Sanz A.B. (2022). Tubular Mitochondrial Dysfunction, Oxidative Stress, and Progression of Chronic Kidney Disease. Antioxidants.

[43] Zhang X., Agborbesong E., Li X. (2021). The Role of Mitochondria in Acute Kidney Injury and Chronic Kidney Disease and Its Therapeutic Potential. Int. J. Mol. Sci..

[44] Ho H.J., Shirakawa H. (2022). Oxidative Stress and Mitochondrial Dysfunction in Chronic Kidney Disease. Cells.

[45] Gamboa J.L., Billings F.T., Bojanowski M.T., Gilliam L.A., Yu C., Roshanravan B., Roberts L.J., Himmelfarb J., Ikizler T.A., Brown N.J. (2016). Mitochondrial dysfunction and oxidative stress in patients with chronic kidney disease. Physiol. Rep..

[46] Kowalczyk P., Sulejczak D., Kleczkowska P., Bukowska-Ośko I., Kucia M., Popiel M., Wietrak E., Kramkowski K., Wrzosek K., Kaczyńska K. (2021). Mitochondrial Oxidative Stress-A Causative Factor and Therapeutic Target in Many Diseases. Int. J. Mol. Sci..

[47] Srivastava A., Tomar B., Sharma D., Rath S.K. (2023). Mitochondrial dysfunction and oxidative stress: Role in chronic kidney disease. Life Sci..

[48] Verma S., Singh P., Khurana S., Ganguly N.K., Kukreti R., Saso L., Rana D.S., Taneja V., Bhargava V. (2021). Implications of oxidative stress in chronic kidney disease: A review on current concepts and therapies. Kidney Res. Clin. Pract..

[49] Krata N., Zagożdżon R., Foroncewicz B., Mucha K. (2018). Oxidative Stress in Kidney Diseases: The Cause or the Consequence?. Arch. Immunol. Ther. Exp..

[50] Düsing P., Zietzer A., Goody P.R., Hosen M.R., Kurts C., Nickenig G., Jansen F. (2021). Vascular pathologies in chronic kidney disease: Pathophysiological mechanisms and novel therapeutic approaches. J. Mol. Med..

[51] Nuhu F., Bhandari S. (2018). Oxidative Stress and Cardiovascular Complications in Chronic Kidney Disease, the Impact of Anaemia. Pharmaceuticals.

[52] Hanna R.M., Streja E., Kalantar-Zadeh K. (2021). Burden of Anemia in Chronic Kidney Disease: Beyond Erythropoietin. Adv. Ther..

[53] Kravvariti E., Ntouros P.A., Vlachogiannis N.I., Pappa M., Souliotis V.L., Sfikakis P.P. (2023). Geriatric Frailty Is Associated with Oxidative Stress, Accumulation, and Defective Repair of DNA Double-Strand Breaks Independently of Age and Comorbidities. J. Gerontol. A Biol. Sci. Med. Sci..

[54] Liu Y. (2011). Cellular and molecular mechanisms of renal fibrosis. Nat. Rev. Nephrol..

[55] Niculae A., Gherghina M.E., Peride I., Tiglis M., Nechita A.M., Checherita I.A. (2023). Pathway from Acute Kidney Injury to Chronic Kidney Disease: Molecules Involved in Renal Fibrosis. Int. J. Mol. Sci..

[56] Krishnan S., Suarez-Martinez A.D., Bagher P., Gonzalez A., Liu R., Murfee W.L., Mohandas R. (2021). Microvascular dysfunction and kidney disease: Challenges and opportunities?. Microcirculation.

[57] Li L., Lu M., Peng Y., Huang J., Tang X., Chen J., Li J., Hong X., He M., Fu H. (2023). Oxidatively stressed extracellular microenvironment drives fibroblast activation and kidney fibrosis. Redox Biol..

[58] Zhou D., Liu Y. (2016). Renal fibrosis in 2015: Understanding the mechanisms of kidney fibrosis. Nat. Rev. Nephrol..

[59] Panizo S., Martínez-Arias L., Alonso-Montes C., Cannata P., Martín-Carro B., Fernández-Martín J.L., Naves-Díaz M., Carrillo-López N., Cannata-Andía J.B. (2021). Fibrosis in Chronic Kidney Disease: Pathogenesis and Consequences. Int. J. Mol. Sci..

[60] Huang R., Fu P., Ma L. (2023). Kidney fibrosis: From mechanisms to therapeutic medicines. Signal Transduct. Target. Ther..

[61] Vanhove T., Goldschmeding R., Kuypers D. (2017). Kidney Fibrosis: Origins and Interventions. Transplantation.

[62] Kaissling B., Lehir M., Kriz W. (2013). Renal epithelial injury and fibrosis. Biochim. Biophys. Acta.

[63] Rayego-Mateos S., Valdivielso J.M. (2020). New therapeutic targets in chronic kidney disease progression and renal fibrosis. Expert Opin. Ther. Targets.

[64] Greco E.V., Russo G., Giandalia A., Viazzi F., Pontremoli R., De Cosmo S. (2019). GLP-1 Receptor Agonists and Kidney Protection. Medicina.

[65] Jastreboff A.M., Kushner R.F. (2023). New Frontiers in Obesity Treatment: GLP-1 and Nascent Nutrient-Stimulated Hormone-Based Therapeutics. Annu. Rev. Med..

[66] Alicic R.Z., Neumiller J.J. (2023). Incretin Therapies for Patients with Type 2 Diabetes and Chronic Kidney Disease. J. Clin. Med..

[67] Drucker D.J. (2022). GLP-1 physiology informs the pharmacotherapy of obesity. Mol. Metab..

[68] Muskiet M.H.A., Tonneijck L., Smits M.M., van Baar M.J.B., Kramer M.H.H., Hoorn E.J., Joles J.A., van Raalte D.H. (2017). GLP-1 and the kidney: From physiology to pharmacology and outcomes in diabetes. Nat. Rev. Nephrol..

[69] Cases A. (2023). Glucagon-like peptide 1 (GLP-1) receptor agonists in the management of the patient with type 2diabetes mellitus and chronic kidney disease: An approach for the nephrologist. Nefrologia.

[70] Winiarska A., Knysak M., Nabrdalik K., Gumprecht J., Stompór T. (2021). Inflammation and Oxidative Stress in Diabetic Kidney Disease: The Targets for SGLT2 Inhibitors and GLP-1 Receptor Agonists. Int. J. Mol. Sci..

[71] Li Y.K., Ma D.X., Wang Z.M., Hu X.F., Li S.L., Tian H.Z., Wang M.J., Shu Y.W., Yang J. (2018). The glucagon-like peptide-1 (GLP-1) analog liraglutide attenuates renal fibrosis. Pharmacol. Res..

[72] de Boer I.H., Khunti K., Sadusky T., Tuttle K.R., Neumiller J.J., Rhee C.M., Rosas S.E., Rossing P., Bakris G. (2022). Diabetes Management in Chronic Kidney Disease: A Consensus Report by the American Diabetes Association (ADA) and Kidney Disease: Improving Global Outcomes (KDIGO). Diabetes Care.

[73] Aldrich S., Ashjian E. (2019). Use of GLP-1 receptor agonists in patients with T2DM and chronic kidney disease. Nurse Pract..

[74] Kristensen S.L., Rørth R., Jhund P.S., Docherty K.F., Sattar N., Preiss D., Køber L., Petrie M.C., McMurray J.J.V. (2019). Cardiovascular, mortality, and kidney outcomes with GLP-1 receptor agonists in patients with type 2 diabetes: A systematic review and meta-analysis of cardiovascular outcome trials. Lancet Diabetes Endocrinol..

[75] Kelly M., Lewis J., Rao H., Carter J., Portillo I., Beuttler R. (2022). Effects of GLP-1 receptor agonists on cardiovascular outcomes in patients with type 2 diabetes and chronic kidney disease: A systematic review and meta-analysis. Pharmacotherapy.

[76] Nabrdalik-Leśniak D., Nabrdalik K., Irlik K., Janota O., Kwiendacz H., Szromek-Białek P., Maziarz M., Stompór T., Gumprecht J., Lip G.Y.H. (2023). The influence of SGLT2 inhibitors on oxidative stress in heart failure and chronic kidney disease in patients with type 2 diabetes. Endokrynol. Pol..

[77] Santulli G., Varzideh F., Forzano I., Wilson S., Salemme L., de Donato A., Lombardi A., Rainone A., Nunziata L., Jankauskas S.S. (2023). Functional and Clinical Importance of SGLT2-inhibitors in Frailty: From the Kidney to the Heart. Hypertension.

[78] Di Costanzo A., Esposito G., Indolfi C., Spaccarotella C.A.M. (2023). SGLT2 Inhibitors: A New Therapeutical Strategy to Improve Clinical Outcomes in Patients with Chronic Kidney Diseases. Int. J. Mol. Sci..

[79] Heerspink H.J.L., Berger S., Gansevoort R.T., Renal Life Cycle Trial Investigators (2023). Will SGLT2 Inhibitors Be Effective and Safe in Patients with Severe CKD, Dialysis, or Kidney Transplantation. Clin. J. Am. Soc. Nephrol..

[80] Blazek O., Bakris G.L. (2023). Slowing the Progression of Diabetic Kidney Disease. Cells.

[81] Dai Z.C., Chen J.X., Zou R., Liang X.B., Tang J.X., Yao C.W. (2023). Role and mechanisms of SGLT-2 inhibitors in the treatment of diabetic kidney disease. Front. Immunol..

[82] Klen J., Dolžan V. (2023). SGLT2 Inhibitors in the Treatment of Diabetic Kidney Disease: More than Just Glucose Regulation. Pharmaceutics.

[83] Guo W., Li H., Li Y., Kong W. (2023). Renal intrinsic cells remodeling in diabetic kidney disease and the regulatory effects of SGLT2 Inhibitors. Biomed. Pharmacother..

[84] Layton A.T., Vallon V. (2023). Did you know how SGLT2 inhibitors protect the kidney?. Acta Physiol..

[85] Albakr R.B., Sridhar V.S., Cherney D.Z.I. (2023). Novel Therapies in Diabetic Kidney Disease and Risk of Hyperkalemia: A Review of the Evidence From Clinical Trials. Am. J. Kidney Dis..

[86] Fernández-Fernandez B., Sarafidis P., Soler M.J., Ortiz A. (2023). EMPA-KIDNEY: Expanding the range of kidney protection by SGLT2 inhibitors. Clin. Kidney J..

[87] González-Juanatey J.R., Górriz J.L., Ortiz A., Valle A., Soler M.J., Facila L. (2023). Cardiorenal benefits of finerenone: Protecting kidney and heart. Ann. Med..

[88] (2023). Finerenone for chronic kidney disease associated with type 2 diabetes with albuminuria. Aust. Prescr..

[89] Singh A.K., Singh A., Singh R., Misra A. (2022). Finerenone in diabetic kidney disease: A systematic review and critical appraisal. Diabetes Metab. Syndr..

[90] Lerma E., White W.B., Bakris G. (2023). Effectiveness of nonsteroidal mineralocorticoid receptor antagonists in patients with diabetic kidney disease. Postgrad. Med..

[91] Fujii W., Shibata S. (2023). Mineralocorticoid Receptor Antagonists for Preventing Chronic Kidney Disease Progression: Current Evidence and Future Challenges. Int. J. Mol. Sci..

[92] Agarwal R., Filippatos G., Pitt B., Anker S.D., Rossing P., Joseph A., Kolkhof P., Nowack C., Gebel M., Ruilope L.M. (2022). Cardiovascular and kidney outcomes with finerenone in patients with type 2 diabetes and chronic kidney disease: The FIDELITY pooled analysis. Eur. Heart J..

[93] Di Lullo L., Lavalle C., Scatena A., Mariani M.V., Ronco C., Bellasi A. (2023). Finerenone: Questions and Answers-The Four Fundamental Arguments on the New-Born Promising Non-Steroidal Mineralocorticoid Receptor Antagonist. J. Clin. Med..

[94] Heinig R., Eissing T. (2023). The Pharmacokinetics of the Nonsteroidal Mineralocorticoid Receptor Antagonist Finerenone. Clin. Pharmacokinet..

[95] Eissing T., Goulooze S.C., van den Berg P., van Noort M., Ruppert M., Snelder N., Garmann D., Lippert J., Heinig R., Brinker M. (2024). Pharmacokinetics and pharmacodynamics of finerenone in patients with chronic kidney disease and type 2 diabetes: Insights based on FIGARO-DKD and FIDELIO-DKD. Diabetes Obes. Metab..

[96] Górriz J.L., González-Juanatey J.R., Facila L., Soler M.J., Valle A., Ortiz A. (2023). Finerenone: Towards a holistic therapeutic approach to patients with diabetic kidney disease. Nefrologia.

[97] Patera F., Gatticchi L., Cellini B., Chiasserini D., Reboldi G. (2024). Kidney Fibrosis and Oxidative Stress: From Molecular Pathways to New Pharmaco-logical Opportunities. Biomolecules.

[98] Dhimolea E. (2010). Canakinumab. mAbs.

[99] Dhorepatil A., Ball S., Ghosh R.K., Kondapaneni M., Lavie C.J. (2019). Canakinumab: Promises and Future in Cardiometabolic Diseases and Malignancy. Am. J. Med..

[100] Afsar B., Covic A., Ortiz A., Afsar R.E., Kanbay M. (2018). The Future of IL-1 Targeting in Kidney Disease. Drugs.

[101] Ridker P.M., Everett B.M., Thuren T., MacFadyen J.G., Chang W.H., Ballantyne C., Fonseca F., Nicolau J., Koenig W., Anker S.D. (2017). Antiinflammatory Therapy with Canakinumab for Atherosclerotic Disease. N. Engl. J. Med..

[102] Ridker P.M., MacFadyen J.G., Glynn R.J., Koenig W., Libby P., Everett B.M., Lefkowitz M., Thuren T., Cornel J.H. (2018). Inhibition of Interleukin-1β by Canakinumab and Cardiovascular Outcomes in Patients with Chronic Kidney Disease. J. Am. Coll. Cardiol..

[103] Aday A.W., Ridker P.M. (2018). Antiinflammatory Therapy in Clinical Care: The CANTOS Trial and Beyond. Front. Cardiovasc. Med..

[104] Cherney D.Z.I., Lytvyn Y., McCullough P.A. (2018). Cardiovascular Risk Reduction in Patients with Chronic Kidney Disease: Potential for Targeting Inflammation with Canakinumab. J. Am. Coll. Cardiol..

[105] Ridker P.M., Rane M. (2021). Interleukin-6 Signaling and Anti-Interleukin-6 Therapeutics in Cardiovascular Disease. Circ. Res..

[106] Shah S.R., Abbasi Z., Fatima M., Ochani R.K., Shahnawaz W., Asim Khan M., Shah S.A. (2018). Canakinumab and cardiovascular outcomes: Results of the CANTOS trial. J. Community Hosp. Intern. Med. Perspect..

[107] Ortega-Paz L., Capodanno D., Angiolillo D.J. (2021). Canakinumab for secondary prevention of coronary artery disease. Future Cardiol..

[108] Sfriso P., Bindoli S., Doria A., Feist E., Galozzi P. (2020). Canakinumab for the treatment of adult-onset Still’s disease. Expert Rev. Clin. Immunol..

[109] Efthimiou P., Kontzias A., Hur P., Rodha K., Ramakrishna G.S., Nakasato P. (2021). Adult-onset Still’s disease in focus: Clinical manifestations, diagnosis, treatment, and unmet needs in the era of targeted therapies. Semin. Arthritis Rheum..

[110] Lima-Posada I., Fontana F., Pérez-Villalva R., Berman-Parks N., Bobadilla N.A. (2019). Pirfenidone prevents acute kidney injury in the rat. BMC Nephrol..

[111] Sartiani L., Bartolucci G., Pallecchi M., Spinelli V., Cerbai E. (2022). Pharmacological basis of the antifibrotic effects of pirfenidone: Mechanistic in-sights from cardiac in-vitro and in-vivo models. Front. Cardiovasc. Med..

[112] Bai X., Nie P., Lou Y., Zhu Y., Jiang S., Li B., Luo P. (2021). Pirfenidone is a renal protective drug: Mechanisms, signalling pathways, and preclinical evidence. Eur. J. Pharmacol..

[113] Swaminathan S., Shah S.V. (2008). Novel approaches targeted toward oxidative stress for the treatment of chronic kidney disease. Curr. Opin. Nephrol. Hypertens..

[114] Chávez-Iñiguez J.S., Poo J.L., Ibarra-Estrada M., García-Benavides L., Navarro-Blackaller G., Cervantes-Sánchez C., Nungaray-Pacheco E., Medina-González R., Armendariz-Borunda J., García-García G. (2021). Effect of Prolonged-Release Pirfenidone on Renal Function in Septic Acute Kidney Injury Patients: A Double-Blind Placebo-Controlled Clinical Trial. Int. J. Nephrol..

[115] Hubers S.A., Brown N.J. (2016). Combined Angiotensin Receptor Antagonism and Neprilysin Inhibition. Circulation.

[116] McMurray J.J., Packer M., Desai A.S., Gong J., Lefkowitz M.P., Rizkala A.R., Rouleau J.L., Shi V.C., Solomon S.D., Swedberg K. (2014). Angiotensin-neprilysin inhibition versus enalapril in heart failure. N. Engl. J. Med..

[117] Yusuf S., Teo K.K., Pogue J., Dyal L., Copland I., Schumacher H., Dagenais G., Sleight P., Anderson C., ONTARGET Investigators (2008). Telmisartan, ramipril, or both in patients at high risk for vascular events. N. Engl. J. Med..

[118] Pontremoli R., Borghi C., Perrone Filardi P. (2021). Renal protection in chronic heart failure: Focus on sacubitril/valsartan. Eur. Heart J. Cardiovasc. Pharmacother..

[119] Desai A.S., McMurray J.J., Packer M., Swedberg K., Rouleau J.L., Chen F., Gong J., Rizkala A.R., Brahimi A., Claggett B. (2015). Effect of the angiotensin-receptor-neprilysin inhibitor LCZ696 compared with enalapril on mode of death in heart failure patients. Eur. Heart J..

[120] Solomon S.D., McMurray J.J.V., Anand I.S., Ge J., Lam C.S.P., Maggioni A.P., Martinez F., Packer M., Pfeffer M.A., Pieske B. (2019). Angiotensin-Neprilysin Inhibition in Heart Failure with Preserved Ejection Fraction. N. Engl. J. Med..

[121] Vanholder R., Pletinck A., Schepers E., Glorieux G. (2018). Biochemical and Clinical Impact of Organic Uremic Retention Solutes: A Comprehensive Update. Toxins.

[122] Sedeek M., Nasrallah R., Touyz R.M., Hébert R.L. (2013). NADPH oxidases, reactive oxygen species, and the kidney: Friend and foe. J. Am. Soc. Nephrol..

[123] Zeisberg M., Neilson E.G. (2010). Mechanisms of tubulointerstitial fibrosis. J. Am. Soc. Nephrol..

[124] Kang H., Zhang J., Zhang X., Qin G., Wang K., Deng Z., Fang Y., Chen G. (2020). Effects of sacubitril/valsartan in patients with heart failure and chronic kidney disease: A meta-analysis. Eur. J. Pharmacol..

[125] Judge P.K., Haynes R. (2021). TaleNeprilysin and Neprilysin inhibition in chronic kidney disease. Curr. Opin. Nephrol. Hypertens..

